# High prevalence of breast arterial calcification in pseudoxanthoma elasticum (PXE) – A nationwide study in the Netherlands

**DOI:** 10.1177/1358863X241268872

**Published:** 2024-08-21

**Authors:** Iris M Harmsen, Madeleine Kok, Frank L Visseren, Wilko Spiering, Pim A de Jong

**Affiliations:** 1 Department of Vascular Medicine, University Medical Center Utrecht, Utrecht University, Utrecht, the Netherlands; 2 Department of Radiology and Nuclear Medicine, Rijnstate Hospital, Arnhem, the Netherlands; 3 Department of Radiology, University Medical Center Utrecht, Utrecht University, Utrecht, the Netherlands

**Keywords:** breast arterial calcification, calcification, mammography, pseudoxanthoma elasticum (PXE)

Pseudoxanthoma elasticum (PXE) is a rare metabolic disease caused by mutations in the *ABCC6* gene, presenting with ectopic calcification because of a relative deficit in plasma pyrophosphate.^1^ One of the key manifestations is early development of arterial calcification, resulting in a higher risk of cardiovascular events. The prevalence and extent of arterial calcification on computed tomography (CT) images in PXE has been compared to hospital controls.^2^ Though CT is preferred for quantification of arterial calcification, the prevalence of arterial calcification on CT in the general population is unknown.

Breast arterial calcification (BAC) on mammography provides a unique opportunity to compare the prevalence of arterial calcification in PXE to the general population because the prevalence of BAC in the general population has been widely studied.^3^ Secondly, as BAC is regarded to be located solely in the tunica media,^4^ assessing BAC in patients with PXE can aid us in understanding whether arterial calcification in PXE is more likely to be intimal arterial calcification or medial calcification.

Patients were derived from the UMC Utrecht Expertise Center for PXE (UCEP), treating 365 patients with PXE (September 2023). Women participating in the breast cancer screening program were included in this study (*n* = 125); only eight women over the age of 50 had no mammograms available. The study was approved by the Institutional Review Board of the UMC Utrecht. Written informed consent was obtained from all participants.

BAC was assessed visually on digital mammograms by two independent radiologists, and was classified as absent, single-vessel, or multivessel, in the most severely affected breast (κ = 0.89). Other variables were gathered from medical records following visits to the UCEP: age, history of cardiovascular event, smoking, diabetes, medication use, body mass index (BMI), blood pressure, lipid levels, and kidney function.

The prevalence of BAC was compared to the general population, by including prevalence data, with calculated 95% CI, from studies that have assessed the prevalence in several general population cohorts; for references see supplementary material. Additionally, determinants of BAC in PXE have been assessed with multinomial and binary regression models adjusted for age.

Of the 125 patients included in the study, 103 (82.4%) had BAC, of which 56 (44.8%) subjects had single-vessel BAC and 47 (37.6%) subjects had multivessel BAC. The age trends of BAC prevalence against general population data from the literature is presented in Figure 1. The results of the multinomial and logistic regression models show that multivessel BAC in PXE was associated with older age (odds ratio [OR] 1.55 [1.04–2.30]), lower estimated glomerular filtration rate (eGFR) (OR 1.28 [0.99−1.64]), and diabetes mellitus (OR 13.01 [1.40–120.73]). Other variables (history of cardiovascular event, smoking, medication use, BMI, blood pressure, and lipid levels) were not significantly associated with BAC.

**Figure 1.**
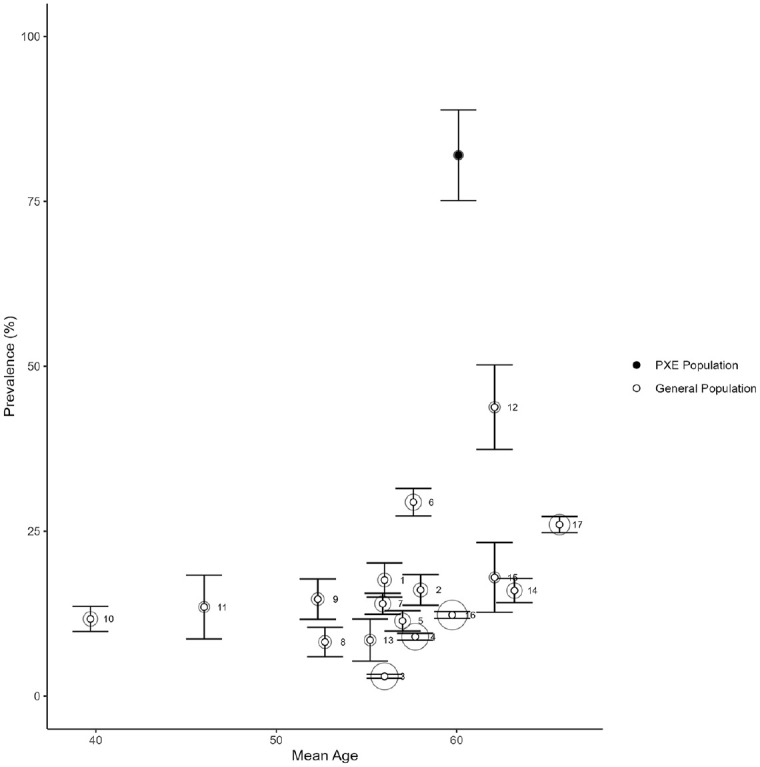
Prevalence of BAC according to mean age in general population studies from the literature and patients with PXE from the current study. Error bars show the 95% CI. White circles: general population studies from the literature; black circles: patients with PXE from the current study. The circle diameters indicate the population size: larger circles represent a larger population. References belonging to the general population studies (1–17) can be found in the supplementary material. BAC, breast arterial calcification; PXE, pseudoxanthoma elasticum. Note: The full size version of this figure is available online.

This nationwide cohort study shows a high involvement of the breast artery in women with PXE over 50 years old, posing a stark contrast to the prevalence of 12.7% reported in a previous meta-analysis of general populations.^3^ The prevalence of BAC increases sharply with age, but even after taking age into account, the prevalence of BAC in patients with PXE is remarkably higher than in the general population.

This result underscores the high calcification burden expected in PXE. Secondly, because BAC is regarded to be present solely in the tunica media, our data support the evidence that the tunica media is involved in PXE, on which there is still debate. The fact that age, kidney function, and diabetes mellitus – the classical risk factors for medial arterial disease – are also risk factors of BAC in PXE, supports this.

A limitation to this study is the categorical approach to the quantification of BAC; a continuous scale would be preferable to minimize the loss of data. Another limitation was a temporal difference between covariates and a mammogram of more than 2 years. To limit bias in these cases, we used multiple imputed measures instead.

In our cohort study, BAC in women with PXE was highly prevalent, which underlines the large calcification burden in PXE. The involvement of the breast artery provides support for medial arterial involvement in PXE and we found increasing age, lower eGFR, and diabetes mellitus to be risk factors for BAC in PXE.

## Supplemental Material

sj-pdf-1-vmj-10.1177_1358863X241268872 – Supplemental material for High prevalence of breast arterial calcification in pseudoxanthoma elasticum (PXE) – A nationwide study in the Netherlands Supplemental material, sj-pdf-1-vmj-10.1177_1358863X241268872 for High prevalence of breast arterial calcification in pseudoxanthoma elasticum (PXE) – A nationwide study in the Netherlands by Iris M Harmsen, Madeleine Kok, Frank L Visseren, Wilko Spiering and Pim A de Jong in Vascular Medicine
