# Supplementary material for: High prevalence of breast arterial calcification in pseudoxanthoma elasticum (PXE) – A nationwide study in the Netherlands
Source: Vasc Med. 2024 Aug 21;29(6):716–7. doi: 10.1177/1358863X241268872 (PMC11590383; doi:10.1177/1358863X241268872)
Supplement: sj-pdf-1-vmj-10.1177_1358863X241268872 – Supplemental material for High prevalence of breast arterial calcification in pseudoxanthoma elasticum (PXE) – A nationwide study in the Netherlands [file sj-pdf-1-vmj-10.1177_1358863X241268872.pdf]

Supplemental References

In this supplement the references are presented, belonging to "**Figure 1.** Prevalence of BAC in PXE versus published general population estimates of BAC prevalence." .

Studies: 1[1] ; 2[2]; 3[3]; 4[4,5]; 5[6]; 6[7]; 7[8,9]; 8[10]; 9[11]; 10[12]; 11[13]; 12[14]; 13[15]; 14[16]; 15[17]; 16[18]; 17[19].

1. Crystal, P., Crystal, E., Leor, J.*, et al.* Breast artery calcium on routine mammography as a potential marker for increased risk of cardiovascular disease. *The American Journal of Cardiology* **86**, 216-217 (2000).
2. Dale, P.S., Mascarhenas, C., Richards, M. & Mackie, G. Mammography as a Screening Tool for Coronary Artery Disease. *Journal of Surgical Research* **148**, 1-6 (2008).
3. Iribarren, C., Go, A.S., Tolstykh, I.*, et al.* Breast Vascular Calcification and Risk of Coronary Heart Disease, Stroke, and Heart Failure. *Journal of Women's Health* **13**, 381-389 (2004).
4. Kemmeren, J.M., van Noord, P.A.H., Beijerinck, D.*, et al.* Arterial Calcification Found on Breast Cancer Screening Mammograms and Cardiovascular Mortality in Women: The DOM Project. *American Journal of Epidemiology* **147**, 333-341 (1998).
5. van Noord, P.A.H., Beijerinck, D., Kemmeren, J.M. & van der Graaf, Y. Mammograms may convey more than breast cancer risk: breast arterial calcification and arterio-sclerotic related diseases in women of the DOM cohort. *European Journal of Cancer Prevention* **5**, 483-487 (1996).
6. Maas, A.H., van der Schouw, Y.T., Beijerinck, D.*, et al.* Arterial calcifications seen on mammograms: cardiovascular risk factors, pregnancy, and lactation. *Radiology* **240**, 33-38 (2006).
7. Reddy, J., Bilezikian, J.P., Smith, S.J. & Mosca, L. Reduced Bone Mineral Density Is Associated with Breast Arterial Calcification. *The Journal of Clinical Endocrinology & Metabolism* **93**, 208-211 (2008).
8. Rotter, M.A., Schnatz, P.F., Currier, A.A., Jr. & O'Sullivan, D.M. Breast arterial calcifications (BACs) found on screening mammography and their association with cardiovascular disease. *Menopause* **15**, 276-281 (2008).
9. Schnatz, P.F., Marakovits, K.A. & O'Sullivan, D.M. The association of breast arterial calcification and coronary heart disease. *Obstet Gynecol* **117**, 233-241 (2011).
10. Sánchez Vidal, M.T., Rodríguez Díaz, J.C., García Pravia, P. & Vizoso Piñeiro, F. Calcificaciones arteriales detectadas en las mamografías. *Revista Clínica Española* **200**, 48 (2000).
11. Sedighi, N., Radmard, A.R., Radmehr, A.*, et al.* Breast arterial calcification and risk of carotid atherosclerosis: Focusing on the preferentially affected layer of the vessel wall. *European Journal of Radiology* **79**, 250-256 (2011).
12. Xue, S., Shen, D., Gao, H. & Wang, Y. Simple Obesity Is Associated with Reduced Breast Arterial Calcification and Increased Plasma Osteopontin Level. *Archives of Medical Research* **39**, 607-609 (2008).
13. Zafar, A.N., Khan, S. & Zafar, S.N. Factors associated with breast arterial calcification on mammography. *J Coll Physicians Surg Pak* **23**, 178-181 (2013).
14. Bielak, L.F., Whaley, D.H., Sheedy, P.F., 2nd & Peyser, P.A. Breast arterial calcification is associated with reproductive factors in asymptomatic postmenopausal women. *J Womens Health (Larchmt)* **19**, 1721-1726 (2010).
15. Ferreira, J.A., Pompei, L.M., Fernandes, C.E., Azevedo, L.H. & Peixoto, S. Breast arterial calcification is a predictive factor of cardiovascular disease in Brazilian postmenopausal women. *Climacteric* **12**, 439-444 (2009).
16. Kataoka, M., Warren, R., Luben, R.*, et al.* How predictive is breast arterial calcification of cardiovascular disease and risk factors when found at screening mammography? *AJR Am J Roentgenol* **187**, 73-80 (2006).

17. Nasser, E.J., Iglésias, E.R., Ferreira, J.A., Fernandes, C.E. & Pompei, L.M. Association of breast vascular calcifications with low bone mass in postmenopausal women. *Climacteric* **17**, 486-491 (2014).
18. Montgomery, G.H., Schnur, J.B., Erblich, J.*, et al.* Breast arterial calcification rates in a diverse, urban group of screening mammography patients. *Annals of Epidemiology* **75**, 16-20 (2022).
19. Iribarren, C., Sanchez, G., Husson, G.*, et al.* MultIethNic Study of BrEast ARterial Calcium Gradation and CardioVAscular Disease: cohort recruitment and baseline characteristics. *Ann Epidemiol* **28**, 41-47.e12 (2018).
